# Association Between Paternal Separation During Early Childhood and Pubertal Timing Among Girls Using Longitudinal Birth Cohort in Japan

**DOI:** 10.3389/fendo.2021.766728

**Published:** 2021-12-21

**Authors:** Aomi Katagiri, Nobutoshi Nawa, Takeo Fujiwara

**Affiliations:** ^1^ Department of Global Health Promotion, Tokyo Medical and Dental University, Tokyo, Japan; ^2^ Department of Medical Education Research and Development, Tokyo Medical and Dental University, Tokyo, Japan

**Keywords:** Paternal separation, paternal absence, puberty, age at peak height velocity, Japan, social epidemiology

## Abstract

**Introduction:**

Previous studies have shown that paternal absence leads to earlier pubertal timing among girls in high-income countries. Despite the low divorce rate in Japan, paternal separation is commonly seen due to a unique corporation system, *tanshin funin*, where employees relocate with their spouses and children. We examined paternal separation, including paternal absence (due to divorce or paternal death) and paternal *tanshin funin*, during early childhood as a predictor of earlier girl’s pubertal development, assessed as age at peak height velocity (PHV).

**Methods:**

This study examined 15 214 girls from a longitudinal survey conducted in Japan from 2001 to 2016 by the Ministry of Health, Labor and Welfare. Paternal separation was determined by the occurrence through annual surveys conducted at ages 0.5 to 4.5 years. Outcome was defined as age at PHV between ages 6 to 15 years. We conducted linear regression, adjusted for potential confounders and other covariates.

**Results:**

Continuous father cohabitation was seen in 88.7% of households, while paternal separation was experienced 1-2, 3-4 and 5 times (always) among 7.4%, 2.8% and 1.1% of households, respectively. Girls who confronted continuous paternal separation (5 times) experienced 0.42 years earlier [95% confidence interval (CI): -0.75, -0.10] age at PHV compared to their peers who always lived with their fathers.

**Conclusion:**

Girls who experienced paternal separation throughout ages 0.5 to 4.5 years experienced PHV earlier.

## Introduction

Earlier pubertal timing in girls has been linked to higher risk of breast cancer (1, 2) and ovarian cancer (2, 3) due to the earlier start of undifferentiated breast cell growth and increased number of ovulations. In addition, early pubertal timing in girls also has an increased risk for psychological dysfunction (e.g., depression and anxiety) (2). These associations took potential confounders into account such as parental socioeconomic status (SES), and early childhood disadvantage. Early pubertal timing is determined by biological factors (4), social factors [such as low family income, low parental education level, absence of biological father (5), stepfather presence (2, 5), family conflict (2), maltreatment (6), urban residency (5)], perinatal factors [such as small for gestational age (7) and maternal overweight during pregnancy (8)], and high BMI before puberty (2).

While parental divorce and father death is an obvious stressor, we hypothesize that physical paternal absence regardless of marital status may also create stress in the household. In Japan, the divorce rate remain low compared to other advanced countries (9). However, there are many more girls who face paternal separation due to *tanshin funin*. *Tanshin funin* is a unique Japanese corporate system in which an employee is transferred to a distant office without his or her family. Nearly 54% of employees in Japan who have a family chose *tanshin funin (* 10), whereas in Western countries families tend to relocate together (11). The households that experience *tanshin funin* are not economically disadvantaged, as they received remittances from the father, and thus differ from the single female households that have been studied in previous studies. However, the physical absence of a father might still affect pubertal timing due to both the physical paternal absence and functional absence having an impact on the mother’s stress level, which in turn results in increased anxiety over the children (11).

Several studies have examined the association between paternal absence and pubertal timing among girls (4, 12, 13). Of note, paternal absence that occurs between the first five years of life (from birth to the age of five) shows stronger association with earlier maturation compared to that occurring in the subsequent five years of life (ages 6-10 years old) (4). The importance of exposure during infancy and early childhood is also consistent with evolutionary theory (14). Although some recent studies found that paternal absence is not associated with early pubertal timing in low to middle income countries, there is consensus that such association remains in high-income countries (15). The variations of paternal separation until the age of five span across a range, in terms of cumulative duration, instability and onset. However, to our knowledge no studies have examined how variations in these patterns might affect pubertal timing in girls. The Japanese society presents an interesting setting to test the hypothesis that paternal separation is correlated with accelerated maturation.

Accordingly, the objectives of this study are to assess the effects of 1) cumulative paternal separation 2) instability of paternal separation and 3) onset of continual paternal separation on girl’s pubertal timing in Japan. Instability of paternal separation can be regarded as stress because such girls may feel more stressed compared to the reference group of girls whose fathers are always present.

## Methods

### Sample

This study used de-identified data from the Longitudinal Survey of Newborns in the 21st Century, collected by the Ministry of Health, Labor and Welfare in Japan from 2001 to 2016 (16). All babies born between January 10th to 17th and July 10th to 17th in 2001 were identified from the national register (n=53 575). Questionnaires were sent *via* postal mail when children were 0.5, 1.5, 2.5, 3.5, 4.5, 5.5, 7, 8, 9, 10, 11, 12, 13, 14, 15 years old, and all data were parent- or self-reported. Participants who did not reply for two years in a row were regarded as dropout. At age 0.5 years, 47 015 children participated.

**Figure 1:**
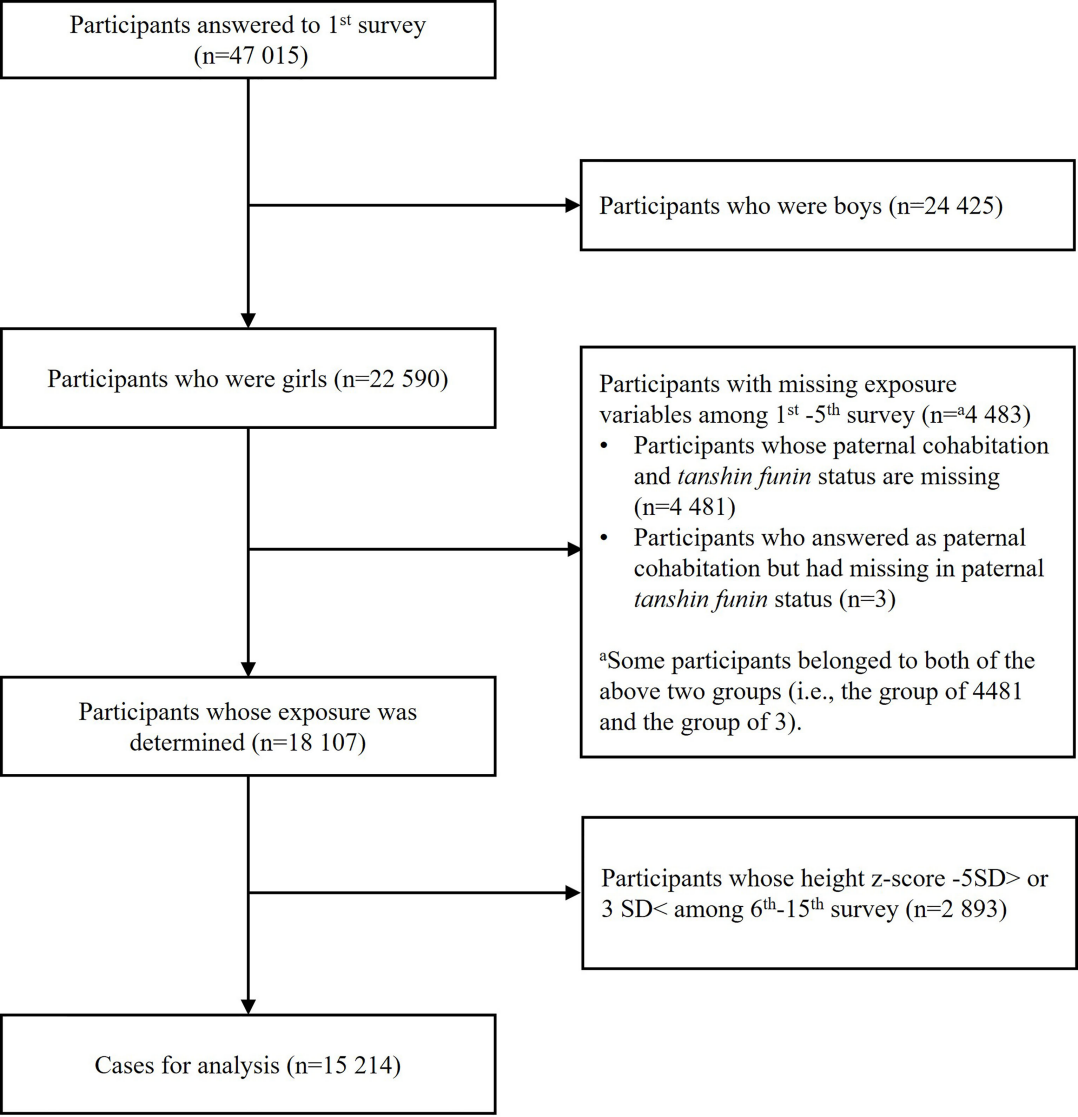
Enrollment of study participants.

We excluded all male participants (n=24 425). After this exclusion, we then excluded participants with missing exposure variables (n=4 483) and participants whose height z-score was less than -5 standard deviation (SD) or larger than 3 SD based on World Health Organization (WHO) standards (n=2 893) (17). This resulted in an analytic sample size of 15 214 girls (**Figure 1**).

A previous profile paper of this cohort showed that this cohort is a representative sample of Japanese children because no difference in the distributions of socio-demographic characteristics such as sex, birthweight, single/multiple births, birth order, birth in/out of wedlock and main employment of the household was observed between responders and the target population (16).

This study was conducted according to the latest guidelines provided in the Declaration of Helsinki and Ministry of Health, Labor and Welfare of Japan and was approved by the ethics committee of Tokyo Medical and Dental University (M2019-066). This study involved a secondary data analysis of de-identified data from the survey by the Ministry of Health, Labor and Welfare in Japan in which consent to participate was assumed upon return of the questionnaires to the Ministry of Health, Labor and Welfare.

### Measurements

Paternal separation included both ‘no father residency’ and ‘on *tanshin funin*’. Father residency was determined from questionnaire responses across survey waves one to five, namely ages 0.5 to 4.5 years. The questionnaire asked responders to mark all members who live with the child and required to include the father if he came back once in three months during *tanshin funin*. Paternal *tanshin funin* was asked in a separate question across waves two to five, however the question was not included in the first survey. Participants who answered ‘no’ to the question about father residency and did not answer the question about *tanshin funin* were categorized as having paternal separation. Participants who answered ‘yes’ to the question about living with their father and did not answer the question about *tanshin funin* were excluded because we could not determine whether they lived with their father or if their father was on *tanshin funin* (n=3). Participants who did not answer the question about living with their father and the question about *tanshin funin* were excluded (n=4 481). There were no participants who did not answer the question about father residency but answered the question about *tanshin funin*. Our main exposure, cumulative occasions of paternal separation, was defined as the sum of occasions when paternal separation was reported, and was categorized as: always living with father, 1-2 occasions of paternal separation, 3-4 occasions of paternal separation, and 5 occasions of paternal separation (n=15 214). Duration of paternal separation may differ by a year at most because the annual questionnaires only asked for the exact time point that the questionnaire was conducted, not the date that the father residency occurred.

Covariates were selected according to previous studies conducted among paternal absence and pubertal timing (4). Covariates included household income at age 0.5, maternal age at first birth, maternal education level and grandparent cohabitation at age 0.5. Regarding maternal age at first birth, lower maternal age at first birth is associated with socioeconomic disadvantages (18) and maternal negative emotions including depression (19). Then, socioeconomic disadvantages (20) and maternal depression are associated with earlier pubertal timing of the child (21). Some researchers also argued that maternal depression leads to attachment difficulties (22) which could accelerate maturation (23). Thus, lower maternal age at first birth could be associated with child’s earlier maturation. Since lower maternal age at first birth has also been linked to single parenthood (4, 18), we regarded maternal age at first birth as a confounder. Household income was included since previous research had pointed out the association with paternal absence (4) and early pubertal growth (5). Grandparent cohabitation was considered because it may lead to paternal absence, and it is also associated with childhood overweight that is associated with earlier maturation (24, 25).

Other covariates were parental smoking status at age 0.5, whether the mother had someone to consult about worries regarding child raising when the child was age 0.5 (i.e., maternal social capital), birth order, rapid weight gain during early childhood, number of siblings at age 4.5, and whether the child was overweight at age 4.5 defined as BMI z score of 1SD or more according to WHO criteria (26). Information on parental smoking status and maternal social capital was from the first survey. These variables were included regarding previous research (27, 28). Rapid weight gain during the first two years of life is linked to earlier pubertal timing (28). Children from father-absent households are more likely to be bottle-fed during infancy, which is associated with rapid weight gain in the first year of life (29).

For maternal age at first birth, we subtracted mother’s birthday from first child’s birthday collected from birth record and first wave. Equivalized household income at age 0.5 was calculated based on responses from first wave. Maternal education level was collected in second wave and categorized as junior high school, high school, vocational school, and higher education. Grandparent cohabitation at age 0.5 was considered when one or more grandparents lived together. Overweight at age 4.5 was determined as BMI z score of 1SD or more according to WHO criteria (26). Rapid weight gain was determined as gaining more than 0.67 SD of weight from birth to age 1.5 years old (28).

Outcome of interest was measured as age at peak height velocity (PHV). Although some prior studies used age at menarche as a measurement for pubertal timing (4, 5, 7), age at PHV has also been used to determine pubertal timing (30–32) and has been known to coincide with decisive pubertal index such as menarche (32–34). We were not able to obtain data for menarche because such question was not included in the survey. Height and date of height measurement were reported by parents, which was shown to be accurate in comparison with objective measurement (35). We first calculated the slope for height growth in each of the two consecutive time points (height at x+1st survey - height at xth survey)/(age at x+1st survey - age at xth survey) and selected the largest slope. Age at PHV was defined as the mean age between the two consecutive time points which the largest height slope was observed [(age at x+1st survey + age at xth survey)/2]. Age at PHV was deemed as missing if it could not be calculated (e.g., when the data on height was missing in either of the two consecutive surveys) (n=1 355).

### Statistical Analysis

We used multiple imputation using chained equations to impute missing values in age at PHV, household income at age 0.5, maternal age at first birth, maternal education level, parental smoking status at age 0.5, maternal social capital when the child was age 0.5, rapid weight gain during early childhood, and whether the child was overweight at age 4.5 and created 20 imputed datasets. Variables used in imputation included cumulative occasions of father separation, birth order and grandparent cohabitation at age 0.5. The results of analyses using all the imputed datasets were combined using Rubin’s rules for multiple imputation (36, 37). Multiple imputation imputed missing valuables for 0.01 to 11.7% of the participants for analysis (n=15 214).

We used linear regression model to estimate the effect of paternal separation on the age at PHV. In our sensitivity analysis, we also examined two alternative ways of assessing paternal separation: instability of paternal separation and commencement of continuous paternal separation. Instability of paternal separation was determined by the number of occasions when the respondents switched between ‘paternal presence’ and ‘paternal separation’ across the five waves (n=15 214). Since the sample was drawn from a population consisting of all babies born in January and July, a sensitivity analysis was also conducted by adjusting for the birth month.

In terms of defining onset of continuous paternal separation, we excluded all children whose fathers later returned and investigated the child’s age when it occurred (n=14 550).

All analyses were performed with STATA SE statistical package, version 14 (StataCorp LP, College Station, ZTX, USA).

## Results

**Table 1** summarizes the characteristics of the sample. Household income at the age of 0.5 years was lower as cumulative occasions of paternal separation increased. Maternal education level was lower in father-always-separated households compared to the others. Also, girls from father-always-separated households were more likely to be an only child, lived with their grandparents during infancy and overweight during early childhood. We also compared our analytic sample with full sample (n=22 590) (**Table Appendix 1**). Characteristics of girls in analytic sample and full sample were similar.

**Table 1:** Characteristics of study participants from the Longitudinal Survey of Newborns in the 21st Century in unimputed original data (n=15214).

Variable		Always father present	1-2 times father separation	3-4 times father separation	Always father separation
		N (%) or mean (SD)	N (%) or mean (SD)	N (%) or mean (SD)	N (%) or mean (SD)
Percentage of participants		13494	1128	420	172
		(88.7)	(7.4)	(2.8)	(1.1)
Age at peak height velocity	Mean, years	9.65	9.67	9.51	9.05
		(2.0)	(2.1)	(2.0)	(2.0)
	Missing	1138	138	49	30
		(8.4)	(12.2)	(11.7)	(17.4)
Household income at age 0.5	Mean, million JPY	2.89	2.66	2.51	1.27
		(1.9)	(1.5)	(1.6)	(1.5)
	Missing	229	14	8	0
		(1.7)	(1.2)	(1.9)	(0.0)
Maternal age at first birth, years	<25	2655	320	137	53
		(19.7)	(28.4)	(32.6)	(30.8)
	25-29	6805	518	186	50
		(50.4)	(45.9)	(44.3)	(29.1)
	30-34	3284	249	84	43
		(24.3)	(22.1)	(20.0)	(25.0)
	35-39	688	37	11	19
		(5.1)	(3.3)	(2.6)	(11.1)
	≧40	61	4	2	7
		(0.5)	(0.4)	(0.5)	(4.1)
	Missing	1	0	0	0
		(0.01)	(0.0)	(0.0)	(0.0)
Maternal education	Junior high school	404	45	26	27
		(3.0)	(4.0)	(6.2)	(15.7)
	High school	5100	432	178	78
		(37.8)	(38.3)	(42.4)	(45.4)
	Vocational school	5817	478	165	56
		(43.1)	(42.4)	(39.3)	(32.6)
	Higher education	2094	170	47	10
		(15.5)	(15.1)	(11.2)	(5.8)
	Others	16	1	0	0
		(0.1)	(0.1)	(0.0)	(0.0)
	Missing	63	2	4	1
		(0.5)	(0.2)	(1.0)	(0.6)
Grandparent cohabitation at age 0.5	Yes	2770	256	110	108
		(20.5)	(22.7)	(26.2)	(62.8)
	No	10724	872	310	64
		(79.5)	(77.3)	(73.8)	(37.2)
Maternal smoking status at age 0.5	Yes	1766	194	110	53
		(13.1)	(17.2)	(26.2)	(30.8)
	No	11677	929	307	117
		(86.5)	(82.4)	(73.1)	(68.0)
	Missing	51	5	3	2
		(0.38)	(0.4)	(0.7)	(1.2)
Paternal smoking status at age 0.5	Yes	8022	712	298	18
		(59.5)	(63.1)	(71.0)	(10.5)
	No	5400	386	99	12
		(40.0)	(34.2)	(23.6)	(7.0)
	Missing	72	30	23	142
		(0.5)	(2.7)	(5.5)	(82.6)
Maternal social capital at age 0.5	Yes	13072	1073	406	156
		(96.9)	(95.1)	(96.7)	(90.7)
	No	89	8	3	10
		(0.7)	(0.7)	(0.7)	(5.8)
	Missing	333	47	11	6
		(2.5)	(4.2)	(2.6)	(3.5)
Birth order	First	6710	547	211	115
		(49.7)	(48.5)	(50.2)	(66.9)
	Second	4966	428	142	38
		(36.8)	(38.0)	(33.8)	(22.1)
	Third	1535	135	57	14
		(11.4)	(12.0)	(13.6)	(8.1)
	Fourth or later	283	18	10	5
		(2.1)	(1.6)	(2.4)	(2.9)
Rapid weight gain from birth to age 1.5	No	7725	616	222	81
		(57.3)	(54.6)	(52.9)	(47.1)
	Yes	4622	411	154	64
		(34.3)	(36.4)	(36.7)	(37.2)
	Missing	1147	101	44	27
		(8.5)	(9.0)	(10.5)	(15.7)
Number of siblings at age 4.5	None	2429	239	163	109
		(18.0)	(21.2)	(38.8)	(63.4)
	One	7886	629	171	42
		(58.4)	(55.8)	(40.7)	(24.4)
	Two	2712	227	73	15
		(20.1)	(20.1)	(17.4)	(8.7)
	Three or more	467	33	13	6
		(3.5)	(2.9)	(3.1)	(3.5)
Overweight at age 4.5	Yes	1562	135	53	26
		(11.6)	(12.0)	(12.6)	(15.1)
	No	10391	834	318	120
		(77.0)	(73.9)	(75.7)	(69.8)
	Missing	1541	159	49	26
		(11.4)	(14.1)	(11.7)	(15.1)
Paternal age at first birth, years	<25	1596	169	89	27
		(11.8)	(15.0)	(21.2)	(15.7)
	25-29	5393	448	158	24
		(40.0)	(39.7)	(37.6)	(14.0)
	30-34	4386	337	103	20
		(32.5)	(29.9)	(24.5)	(11.6)
	35-39	1578	119	40	13
		(11.7)	(10.6)	(9.5)	(7.6)
	≧40	516	32	12	9
		(3.8)	(2.8)	(2.9)	(5.2)
	Missing	25	23	18	79
		(0.2)	(2.0)	(4.3)	(45.9)
Paternal education	Junior high school	691	91	57	16
		(5.1)	(8.1)	(13.6)	(9.3)
	High school	5141	452	152	34
		(38.1)	(40.1)	(36.2)	(19.8)
	Vocational school	2347	153	62	14
		(17.4)	(13.6)	(14.8)	(8.1)
	Higher education	5224	418	105	23
		(38.7)	(37.1)	(25.0)	(13.4)
	Others	15	2	1	0
		(0.1)	(0.2)	(0.2)	(0.0)
	Missing	76	12	43	85
		(0.6)	(1.1)	(10.2)	(49.4)
Residential area	20 designated cities	2892	246	96	38
		(21.4)	(21.8)	(22.9)	(22.1)
	Other cities	8070	661	237	101
		(59.8)	(58.6)	(56.4)	(58.7)
	Rural	2532	221	87	33
		(18.8)	(19.6)	(20.7)	(19.2)
Gestational period, weeks	22-36	524	55	26	10
		(3.9)	(4.9)	(6.2)	(5.8)
	37-41	12845	1060	389	160
		(95.2)	(94.0)	(92.6)	(93.0)
	≧42	125	13	5	2
		(0.9)	(1.2)	(1.2)	(1.2)
Birth weight, grams	<2500	1224	102	41	21
		(9.1)	(9.0)	(9.8)	(12.2)
	≧2500	12270	1026	379	151
		(90.9)	(91.0)	(90.2)	(87.8)

**Table 2** shows the association between cumulative occasions of paternal separation from ages 0.5 to 4.5 years and age at PHV using linear regression. After adjusting for potential confounders, girls whose fathers were always separated experienced PHV 0.47 years (95% CI: -0.80, -0.15, p=0.004) earlier compared to girls whose father was always present (Model 1, **Table 2**). However, the intermediate categories of paternal separation (father separated on 1-2 occasions, 3-4 occasions) were not statistically significantly associated with age at PHV.

**Table 2:** Associations between cumulative occasions of father separation at ages 0.5-4.5 and age at peak height velocity (years) using linear regression (n=15214).

		Crude	Model 1	Model 2
		β	β	β
		(95%CI)	(95%CI)	(95%CI)
Always father present		Ref	Ref	Ref
1-2 times father separation		0.002	0.01	0.03
		(-0.13, 0.13)	(‐0.12, 0.15)	(-0.10, 0.16)
3-4 times father separation		-0.14	-0.11	-0.06
		(-0.35, 0.08)	(‐0.32, 0.11)	(-0.28, 0.15)
Always father separation		**-0.58**	**-0.47**	**-0.42**
		**(-0.90, -0.26)**	**(-0.80, -0.15)**	**(-0.75, -0.10)**
				
Household income		**-**	1.98×10^-7^	-6.70×10^-8^
			(-1.73×10^-6^, 2.12×10^-6^)	(-1.99×10^-6^, 1.86×10^-6^)
Maternal age at first birth		**-**	0.01	0.01
			(-0.001, 0.02)	(-0.001, 0.02)
Maternal education level	Junior high school	**-**	Ref	Ref
	High school	**-**	0.16	0.12
			(-0.05, 0.37)	(-0.09, 0.34)
	Vocational school	**-**	**0.23**	0.17
			**(0.02, 0.43)**	(- 0.05, 0.38)
	Higher education	**-**	**0.25**	0.18
			**(0.03, 0.47)**	(-0.04, 0.41)
	Others	**-**	0.02	-0.03
			(-0.98, 1.02)	(-1.04, 0.97)
Grandparent cohabitation at age 0.5	No	**-**	Ref	Ref
	Yes	**-**	**-0.16**	**-0.15**
			**(-0.24, -0.07)**	**(-0.24, -0.06)**
Maternal smoking status at age 0.5	No	**-**	**-**	Ref
	Yes	**-**	**-**	-0.09
				(-0.19, 0.02)
Paternal smoking status at age 0.5	No	**-**	**-**	Ref
	Yes	**-**	**-**	-0.08
				(-0.15, 0.004)
Maternal social capital at age 0.5	Yes	**-**	–	Ref
	No	**-**	–	0.02
				(-0.39, 0.43)
Birth order		**-**	–	-0.06
				(-0.12, 0.001)
Rapid weight gain from birth to age 1.5	No	**-**	–	Ref
	Yes	**-**	–	**-0.10**
				**(-0.18, -0.03)**
Number of siblings at age 4.5		**-**	–	**0.07**
				**(0.004, 0.13)**
Overweight at age 4.5	No	**-**	–	Ref
	Yes	**-**	–	**-0.28**
				**(-0.39, -0.17)**

Bolded values indicate statistical significance at p < 0.05.

Model 1 adjusted for household income, maternal age at first birth, maternal education level and grandparent cohabitation.

Model 2 adjusted for parental smoking status, maternal social capital, birth order, rapid weight gain, number of siblings and childhood overweight in addition to covariates included in Model1.

As a crude check of mediation, after including potential variables, girls who did not live with their father on all five occasions still experienced an earlier onset of PHV of 0.42 years (95% CI: -0.75, -0.10, p=0.011) (Model 2, **Table 2**). We also conducted sensitive analysis by adjusting for birth month as the population derived from those born in January and July. Girls from father-always-separated households still experienced 0.43 years earlier (95% CI: -0.75, -0.10, p=0.010) maturation after adjustment.

Next, we turned to an alternative definition of exposure, that is, instability of paternal separation. The results (Model 1, **Table Appendix 2**) showed that while such instability was correlated with earlier peak velocity, the estimate was not statistically significant. Finally, when we looked at the onset of continuous paternal separation, our results showed that age at PHV comes earlier by 0.42 years when paternal separation was reported at the very first survey (95% CI: -0.74, -0.09, p=0.011). When paternal absence occurred after the age of 0.5, the results were not significant.

## Discussion

This study found that cumulative paternal separation from infancy was associated with a significantly earlier age at PHV in girls. We also found that the cumulative effect from infancy was stronger than that of instability or onset of continual paternal separation at later ages. Previous studies have emphasized that the association between paternal absence and earlier pubertal timing is stronger when it occurs during early childhood (ages 0 through 5 years) than that occurring at later ages (4). We add to the literature the cumulative effect of physical paternal absence including *tanshin funin* on earlier age at PHV among girls.

Several explanations between father absence and earlier maturation have been suggested in previous research. The first possible mechanism linking paternal absence to earlier pubertal growth among girls is low SES and higher risk of overweight. Previous studies have shown that paternal absence is strongly correlated with lower SES (38), because such households rely on the income of a single parent (usually the mother), and women usually earn less compared to men due to the gender wage gap (39). In addition, socioeconomic disadvantage is correlated with a higher risk of obesity in high-income countries where energy-dense food is cheaper and more readily accessible to low-incomed families (40). Furthermore, overweight is associated with earlier pubertal timing due to accelerated gonadal function stemming from increased insulin resistance, androgens, and leptin levels (8, 24). This trend has also been observed in Japan (41) and was also seen in the population we analyzed. As seen in **Table 1**, father-always-absent families had lower household income at the age of 0.5, and girls from such families were more likely to be overweight during early childhood. However, low SES is unlikely to fully explain our findings because earlier maturation was still observed after adjusting for household income and overweight (**Table 2**, Model 2). This can be because girls who experience father absence due to *tanshin funin* did not experience economic disadvantage in same ways as their peers whose father was absent for different reasons (i.e. divorce/death). However, we were not able to differentiate father absence according to the reason because the first survey lacked questions on *tanshin funin*.

An alternative explanation invokes the role of chronic stress and increased sex hormone secretion induced from paternal absence (4, 42). When stress is of a chronic nature, the HPA (hypothalamic-pituitary-adrenal) axis and the HPG (hypothalamic-pituitary-gonadal) axis can become dysregulated, triggering increased secretion of GnRH (gonadotropin-releasing hormone) and downstream gonadotropin hormones (43) that cause early maturation and pubertal timing. In line with this, studies have shown that chronic stress (including paternal absence and parent conflict) is associated with earlier puberty (4). However, in our study, we could not confirm the effect of stress as we did not have such information in the survey. Although other proxy measures of stress such as instability of paternal separation did not show a significant association with earlier pubertal timing, future studies assessing whether stress mediates the association are warranted.

Besides the aforementioned pathways, some researchers have argued for an evolutionary basis for the correlation between social-environmental adversity and early pubertal timing (12, 14, 44). Social adversity (including poverty, parental instability, high stress) during early childhood tends to foster more insecure attachment between parents and children. In turn, insecure attachment may give rise to behavioral problems (externalizing symptoms) and depression/anxiety (internalizing symptoms). Girls are more likely to develop internalizing problems, which correlates with lower metabolism, storage of fat, and menarche stimulation – part of the biological and behavioral “quantity-oriented reproductive strategy” (14). Such a strategy might maximize the chance of survival at the level of the individual within a species in the context of adversity. Future studies should assess whether biological mechanisms (e.g., hormonal changes) that are consistent with the evolutionary theory may explain association between paternal absence and earlier pubertal timing.

Although prior studies in Japanese girls showed similar results as our study on mean age at PHV (45), some had different results (31). This gap may be because our study used data from recent surveys, while prior studies used older data. Studies pointed out that there is a trend of maturation acceleration over the past decades (46). The difference in height cutoff may also explain the difference in age at PHV.

This study has several limitations. First, the survey only included height as a measure of pubertal development and lacked information on age at menarche (4, 5) or breast or pubic hair development (27). Furthermore, height measurement was limited annually. Nonetheless, height is easy to measure, and age at PHV is suggested to coincide with other pubertal index, thus can be applicable to detect signs of early puberty (47, 48) that enables future possible interventions for early maturation. Since it is also difficult to detect puberty-related growth acceleration that occurs prior to its peak, future study should combine other measurements for puberty to confirm our findings, as well as measure height at several points within a year. Second, we could not obtain measurements of stress or hormone levels that might help explain the association between father absence and earlier maturation in terms of chronic stress/increased hormone pathway or the evolutionary theory. Third, we were not able to include pubertal timing of the parents because the survey lacked maternal age of menarche as well as age of paternal puberty. Fourth, we did not have sufficient observation time for the boys in the sample to attain PHV (49). Fifth, we were not able to separately analyze paternal separation resulting from divorce/death and *tanshin funin*. This is because there was no question on *tanshin funin* in the first survey and we could not clarify whether paternal separation was due to *tanshin funin* or divorce/death. Sixth, paternal absence was self-reported. Since the questionnaire only queried father cohabitation at the exact timepoint of survey, the length of paternal separation may differ by a year at most. Seventh, we did not have information on whether the father on *tanshin funin* had contact with the family. Eighth, children’s height information was self-reported from a family member. Although it is possible that there may be inaccurate records, especially in outliers, we consider that our sample was representative of the Japanese population based on a previous cohort profile study (16). Furthermore, characteristics of girls in our analytic sample and full sample were similar under comparison. Lastly, we did not ascertain whether the living father was the biological father (5).

The implication of this study is that girls who experienced cumulative paternal separation from early infancy are at increased risk of earlier puberty and health problems accompanying it. This study adds to the literature that in terms of earlier onset of puberty, not only paternal absence due to divorce/death plays a part but also physical and functional father absence due to *tanshin funin*. Although SES has been considered as an important pathway between paternal absence and earlier maturation, it does not fully explain the association in our study. Future studies should clarify the mechanisms including stress that might explain our findings. Girls who experienced early maturation may be differentiated from peers due to their appearance, and thus have an increased risk of psychological stress (4) in addition to existing stress caused by the family structure. Given the increased risk of experiencing earlier puberty in girls who experienced paternal separation, programs that aim to provide support for pubertal psychological care should also provide comprehensive care to girls and their families who experienced *tanshin funin* as well.

## Conclusion

We found that paternal separation throughout ages 0.5 to 4.5 years leads to earlier age at PHV among girls. This effect was cumulative and was stronger than that of instability or onset of paternal separation at later ages.

## Data Availability Statement

The original contributions presented in the study are included in the article/**Supplementary Material**. Further inquiries can be directed to the corresponding author.

## Ethics Statement

The studies involving human participants were reviewed and approved by the Ethics Committee of Tokyo Medical and Dental University. Written informed consent for participation was not required for this study in accordance with the national legislation and the institutional requirements.

## Author Contributions

Conceptualization, AK, NN, and TF. Methodology, AK and NN. Software, AK. Validation, NN. Formal analysis, AK. Investigation, AK. Resources, TF. Data curation, AK and NN. Writing – original draft preparation, AK. Writing – review and editing, NN. Visualization, AK. Supervision, TF. Project Administration, TF. All authors contributed to the article and approved the submitted version.

## Funding

This study was supported by Grant-in-Aid for Challenging Research (Pioneering) from the Japan Society for the Promotion of Science (Grant Number 21K18294). AK is a recipient of a scholarship by the Japan Student Services Organization.

The funder had no role in study design, data collection and analysis, decision to publish, or preparation of the manuscript.

## Conflict of Interest

The authors declare that the research was conducted in the absence of any commercial or financial relationships that could be construed as a potential conflict of interest.

## Publisher’s Note

All claims expressed in this article are solely those of the authors and do not necessarily represent those of their affiliated organizations, or those of the publisher, the editors and the reviewers. Any product that may be evaluated in this article, or claim that may be made by its manufacturer, is not guaranteed or endorsed by the publisher.
