# Supplementary material for: Association Between Paternal Separation During Early Childhood and Pubertal Timing Among Girls Using Longitudinal Birth Cohort in Japan
Source: Front Endocrinol (Lausanne). 2021 Dec 21;12:766728. doi: 10.3389/fendo.2021.766728 (PMC8724124; doi:10.3389/fendo.2021.766728)
Supplement: Supplementary file 1 [file DataSheet_1.docx]

| Appendix Table 1. Characteristics of study participants from the Longitudinal Survey of Newborns in the 21st Century in original unimputed dataset (n=22590) | | | | | | | | | | |
| --- | --- | --- | --- | --- | --- | --- | --- | --- | --- | --- |
|  |  | **Always father present** |  | **1-2 times father separation** |  | **3-4 times father separation** |  | **Always father separation** |  | **Missing** |
| **Variable** |  | **N (%) or  mean (SD)** |  | **N (%) or  mean (SD)** |  | **N (%) or  mean (SD)** |  | **N (%) or  mean (SD)** |  | **N (%) or  mean (SD)** |
| Number of participants | | 16046 (71.0) |  | 1352 (6.0) |  | 508 (2.3) |  | 203 (0.9) |  | 4481 (19.8) |
| Age at peak height velocity, years | | 9.76 (2.0) |  | 9.81 (2.0) |  | 9.68 (2.0) |  | 9.20 (1.9) |  | 9.26 (2.0) |
| Household income at age 0.5, million JPY | | 2.87 (1.9) |  | 2.65 (1.5) |  | 2.47 (1.6) |  | 1.24 (1.5) |  | 2.23 (1.6) |
| Maternal age at first birth, years | <25 | 3217 (20.1) |  | 386 (28.6) |  | 175 (34.5) |  | 64 (31.5) |  | 1831 (40.9) |
|  | 25-29 | 8082 (50.4) |  | 618 (45.7) |  | 215 (42.3) |  | 63 (31.0) |  | 2147 (39.7) |
|  | 30-34 | 3851 (24.0) |  | 294 (21.8) |  | 103 (20.3) |  | 48 (23.7) |  | 725 (16.2) |
|  | 35-39 | 815 (5.1) |  | 47 (3.5) |  | 13 (2.6) |  | 19 (9.4) |  | 128 (2.9) |
|  | ≧40 | 80 (0.5) |  | 7 (0.5) |  | 2 (0.4) |  | 8 (3.9) |  | 10 (0.2) |
|  | Missing | 1 (0.01) |  | 0 (0.0) |  | 0 (0.0) |  | 1 (0.5) |  | 8 (0.2) |
| Maternal education | Junior high school | 476 (3.0) |  | 50 (3.7) |  | 34 (6.7) |  | 33 (16.3) |  | 301 (6.7) |
|  | High school | 6108 (38.1) |  | 530 (39.2) |  | 217 (42.7) |  | 91 (44.8) |  | 1348 (30.1) |
|  | Vocational school | 6946 (43.3) |  | 573 (42.4) |  | 196 (38.6) |  | 66 (32.5) |  | 1040 (23.2) |
|  | Higher education | 2428 (15.1) |  | 196 (14.5) |  | 56 (11.0) |  | 12 (5.9) |  | 269 (6.0) |
|  | Others | 16 (0.1) |  | 1 (0.1) |  | 0 (0.0) |  | 0 (0.0) |  | 6 (0.1) |
|  | Missing | 72 (0.5) |  | 2 (0.2) |  | 5 (1.0) |  | 1 (0.5) |  | 1517 (33.9) |
| Grandparent cohabitation at age 0.5 | Yes | 3296 (20.5) |  | 320 (23.7) |  | 139 (27.4) |  | 121 (59.6) |  | 1071 (23.9) |
|  | No | 12750 (79.5) |  | 1032 (76.3) |  | 369 (72.6) |  | 82 (40.4) |  | 3410 (76.1) |
| Maternal smoking status at age 0.5 | Yes | 2139 (13.3) |  | 225 (16.6) |  | 138 (27.2) |  | 70 (34.5) |  | 1334 (29.8) |
|  | No | 13845 (86.3) |  | 1120 (82.8) |  | 367 (72.2) |  | 131 (64.5) |  | 3091 (69.0) |
|  | Missing | 62 (0.4) |  | 7 (0.5) |  | 3 (0.6) |  | 2 (1.0) |  | 56 (1.3) |
| Paternal smoking status at age 0.5 | Yes | 9644 (60.1) |  | 848 (62.7) |  | 364 (71.7) |  | 19 (9.4) |  | 3154 (70.4) |
|  | No | 6321 (39.4) |  | 469 (34.7) |  | 116 (22.8) |  | 12 (5.9) |  | 1130 (25.2) |
|  | Missing | 81 (0.5) |  | 35 (2.6) |  | 28 (5.5) |  | 172 (84.7) |  | 197 (4.4) |
| Maternal social capital at age 0.5 | Yes | 15524 (96.8) |  | 1287 (95.2) |  | 489 (96.3) |  | 182 (90.2) |  | 4214 (94.0) |
|  | No | 111 (0.7) |  | 12 (0.9) |  | 6 (1.2) |  | 12 (5.9) |  | 63 (1.4) |
|  | Missing | 411 (2.6) |  | 53 (3.9) |  | 13 (2.6) |  | 8 (3.9) |  | 204 (4.6) |
| Birth order | First | 8105 (50.5) |  | 661 (48.9) |  | 252 (49.6) |  | 133 (65.5) |  | 2287 (51.0) |
|  | Second | 5837 (36.4) |  | 502 (37.1) |  | 178 (35.0) |  | 49 (24.1) |  | 1551 (34.6) |
|  | Third | 1778 (11.1) |  | 161 (11.9) |  | 66 (13.0) |  | 16 (7.9) |  | 479 (10.7) |
|  | Fourth or later | 326 (2.0) |  | 28 (2.1) |  | 12 (2.4) |  | 5 (2.5) |  | 164 (3.7) |
| Rapid weight gain from birth to age 1.5 | No | 9112 (56.8) |  | 735 (654.4) |  | 266 (52.4) |  | 99 (48.8) |  | 1547 (34.5) |
|  | Yes | 5534 (34.5) |  | 491 (36.3) |  | 186 (36.6) |  | 75 (37.0) |  | 976 (21.8) |
|  | Missing | 1400 (8.7) |  | 126 (9.3) |  | 56 (11.0) |  | 29 (14.3) |  | 1958 (43.7) |
| Number of siblings at age 4.5 | None | 2921 (18.2) |  | 295 (21.8) |  | 195 (38.4) |  | 126 (62.1) |  | 241 (5.4) |
|  | One | 9404 (58.6) |  | 740 (54.7) |  | 210 (41.3) |  | 54 (26.6) |  | 504 (11.3) |
|  | Two | 3177 (19.8) |  | 271 (20.0) |  | 87 (17.1) |  | 17 (8.4) |  | 190 (4.2) |
|  | Three or more | 544 (3.4) |  | 46 (3.4) |  | 16 (3.2) |  | 6 (3.0) |  | 58 (1.3) |
|  | Missing | 0 (0.0) |  | 0 (0.0) |  | 0 (0.0) |  | 0 (0.0) |  | 3488 (77.8) |
| Overweight at age 4.5 | Yes | 1852 (11.5) |  | 161 (11.9) |  | 61 (12.0) |  | 30 (14.8) |  | 109 (2.4) |
|  | No | 12097 (75.4) |  | 982 (72.6) |  | 377 (74.2) |  | 136 (67.0) |  | 652 (14.6) |
|  | Missing | 2097 (13.1) |  | 209 (15.5) |  | 70 (13.8) |  | 37 (18.2) |  | 3720 (83.0) |
| Paternal age at first birth, years | <25 | 1935 (12.1) |  | 211 (15.6) |  | 115 (22.6) |  | 30 (14.8) |  | 1164 (26.0) |
|  | 25-29 | 6404 (39.9) |  | 528 (39.1) |  | 184 (36.2) |  | 32 (15.8) |  | 1643 (36.7) |
|  | 30-34 | 5199 (32.4) |  | 409 (30.3) |  | 128 (25.2) |  | 20 (9.9) |  | 988 (22.1) |
|  | 35-39 | 1869 (11.7) |  | 135 (10.0) |  | 44 (8.7) |  | 15 (7.4) |  | 380 (8.5) |
|  | ≧40 | 609 (3.8) |  | 43 (3.2) |  | 14 (2.8) |  | 9 (4.4) |  | 159 (3.6) |
|  | Missing | 30 (0.2) |  | 26 (1.9) |  | 23 (4.5) |  | 97 (47.8) |  | 147 (3.3) |
| Paternal education | Junior high school | 846 (5.3) |  | 108 (8.0) |  | 69 (13.6) |  | 18 (8.9) |  | 393 (8.8) |
|  | High school | 6170 (38.5) |  | 541 (40.0) |  | 184 (36.2) |  | 39 (19.2) |  | 1302 (29.1) |
|  | Vocational school | 2790 (17.4) |  | 190 (14.1) |  | 75 (14.8) |  | 17 (8.4) |  | 449 (10.0) |
|  | Higher education | 6138 (38.3) |  | 499 (36.9) |  | 125 (24.6) |  | 25 (12.3) |  | 749 (16.7) |
|  | Others | 16 (0.1) |  | 2 (0.2) |  | 1 (0.2) |  | 0 (0.0) |  | 1 (0.02) |
|  | Missing | 86 (0.5) |  | 12 (0.9) |  | 54 (10.6) |  | 104 (51.2) |  | 1587 (35.4) |
| Residential area | 20 designated cities | 3462 (21.6) |  | 296 (21.9) |  | 112 (22.1) |  | 48 (23.7) |  | 917 (20.5) |
|  | Other cities | 9597 (59.8) |  | 790 (58.4) |  | 295 (58.1) |  | 120 (59.1) |  | 2695 (60.1) |
|  | Rural | 2987 (18.6) |  | 266 (19.7) |  | 101 (19.9) |  | 35 (17.2) |  | 869 (19.4) |
| Gestational period, weeks | 22-36 | 623 (3.9) |  | 73 (5.4) |  | 27 (5.3) |  | 12 (5.9) |  | 229 (5.1) |
|  | 37-41 | 15276 (95.2) |  | 1266 (93.6) |  | 476 (93.7) |  | 189 (93.1) |  | 4203 (93.8) |
|  | ≧42 | 147 (0.9) |  | 13 (1.0) |  | 5 (1.0) |  | 2 (1.0) |  | 49 (1.1) |
| Birth weight, grams | <2500 | 1449 (9.0) |  | 137 (10.1) |  | 54 (10.6) |  | 24 (11.8) |  | 465 (10.4) |
|  | ≧2500 | 14597 (91.0) |  | 1215 (89.9) |  | 454 (89.4) |  | 179 (88.2) |  | 4016 (89.6) |

| Appendix Table 2. Associations between instability of father separation at ages 0.5-4.5 and age at peak height velocity (years) using linear regression (n=15214) | | | | | | |
| --- | --- | --- | --- | --- | --- | --- |
|  |  | Crude |  | Model 1 |  | Model 2 |
|  |  | β (95%CI) |  | β (95%CI) |  | β (95%CI) |
| Always father present | | Ref |  | Ref |  | Ref |
| Always father separation | | **-0.58 (-0.90, -0.26)** |  | **-0.47 (-0.79, -0.15)** |  | **-0.42 (-0.75, -0.10)** |
| Once | | -0.03 (-0.18, 0.12) |  | -0.01 (-0.16, 0.14) |  | 0.02 (-0.13, 0.17) |
| Twice or more | | -0.05 (-0.23, 0.12) |  | -0.04 (-0.22, 0.14) |  | -0.03 (-0.21, 0.15) |
|  |  |  |  |  |  |  |
| Household income at age 0.5 | | **-** |  | 2.05×10-7 (-1.72×10-6, 2.13×10-6) |  | -6.07×10-8 (-1.99×10-6, 1.86×10-6) |
| Maternal age at first birth | | **-** |  | 0.01 (-0.001, 0.02) |  | 0.01 (-0.001, 0.02) |
| Maternal education level | Junior high school | **-** |  | Ref |  | Ref |
|  | High school | **-** |  | 0.16 (-0.05, 0.37) |  | 0.12 (-0.10, 0.34) |
|  | Vocational school | **-** |  | **0.23 (0.02, 0.44)** |  | 0.17 (-0.05, 0.38) |
|  | Higher education | **-** |  | **0.26 (0.04, 0.48)** |  | 0.18 (-0.04, 0.41) |
|  | Others | **-** |  | 0.02 (-0.98, 1.03) |  | -0.03 (-1.04, 0.97) |
| Grandparent cohabitation at age 0.5 | No | **-** |  | Ref |  | Ref |
|  | Yes | **-** |  | **-0.16 (-0.24, -0.07)** |  | **-0.15 (-0.24, -0.06)** |
| Maternal smoking status | No | **-** |  | **-** |  | Ref |
|  | Yes | **-** |  | **-** |  | -0.09 (-0.20, 0.02) |
| Paternal smoking status | No | **-** |  | **-** |  | Ref |
|  | Yes | **-** |  | **-** |  | **-0.08 (-0.15, -0.005)** |
| Maternal social capital at age 0.5 | Yes | **-** |  | - |  | Ref |
|  | No | **-** |  | - |  | 0.19 (-0.39, 0.43) |
| Birth order | | **-** |  | - |  | **-0.06 (-0.13, -0.001)** |
| Rapid weight gain from birth to age 1.5 | No | **-** |  | - |  | Ref |
|  | Yes | **-** |  | - |  | **-0.10 (-0.18, -0.03)** |
| Number of siblings at age 4.5 |  | **-** |  | - |  | **0.07 (0.01, 0.14)** |
| Overweight at age 4.5 | No | **-** |  | - |  | Ref |
|  | Yes | **-** |  | - |  | **-0.28 (-0.39, -0.17)** |
| Bolded values indicate statistical significance at p<0.05. | | | | | | |
| Model 1 adjusted for household income, maternal age at first birth, maternal education level and grandparent cohabitation. | | | | | | |
| Model 2 adjusted for parental smoking status, maternal social capital, birth order, rapid weight gain, number of siblings and childhood overweight in addition to covariates included in Model1. | | | | | | |
